# Periprocedural Anticoagulation Management of Patients Undergoing Colonoscopy with Polypectomy

**DOI:** 10.1055/s-0044-1787553

**Published:** 2024-06-03

**Authors:** Melissa Chan, Joshua Yoon, Jennifer J. Telford, Chipman T. Drury, Tony Wan

**Affiliations:** 1 Department of Medicine, University of British Columbia, Vancouver, Canada; 2 Division of Gastroenterology, University of British Columbia, Vancouver, Canada; 3 British Columbia Colon Screening Program, BC Cancer, Vancouver, Canada; 4 Division of General Internal Medicine, University of British Columbia, Vancouver, Canada

**Keywords:** colonoscopy, polypectomy, anticoagulation, periprocedural management

## Abstract

**Introduction/Objective** Colonoscopy with polypectomy is an integral component of colorectal cancer screening. There are limited data and consensus on periprocedural anticoagulation management, especially regarding bleeding risk with uninterrupted anticoagulation and thromboembolic risk with interruption. Our aim was to determine the incidence of bleeding and thromboembolic complications among colon screening participants undergoing colonoscopy following implementation of a novel patient care pathway for standardized periprocedural anticoagulation management.

**Methods** We conducted a retrospective study including all participants (age 50–74) on an oral anticoagulant (e.g., vitamin K antagonists, direct oral anticoagulants) referred to the British Columbia Colon Screening Program for colonoscopy following abnormal fecal immunochemical test in a 6-month period (March–August 2022). Data relating to their specific periprocedural anticoagulant management and colonoscopy results including method of polypectomy were obtained. Primary outcomes were major bleeding and arterial or venous thromboembolic events from time of oral anticoagulant interruption until 14 days of postcolonoscopy. Secondary outcomes included nonmajor and minor bleeding, acute coronary syndrome, emergency room visit, hospital admission, and death due to any cause.

**Results** Over the 6-month period, 162 participants completed standardized periprocedural anticoagulation management, colonoscopy ± polypectomy, and 14-day follow-up. One (0.6%) had a major bleeding event and one (0.6%) had an arterial thromboembolic event.

**Conclusions** A novel patient care pathway for standardized periprocedural anticoagulation management with a multidisciplinary team is associated with low rates of major bleeding and thrombotic complications after colonoscopy with polypectomy.

## Introduction

Colonoscopy with polypectomy is an integral part of colorectal cancer (CRC) screening both as a primary screening test and to follow-up an abnormal noninvasive screening test, such as the fecal immunochemical test (FIT). 1 Bleeding is the most common complication after colonoscopy occurring in 0.3% of patients. 2 3 Individuals on anticoagulants are at increased risk of bleeding and additional risk factors including bridging anticoagulation with low molecular weight heparin (LMWH), hot snare polypectomy, and large polyp removed further exacerbate this risk. 4 Periprocedural anticoagulation management is controversial due to the limited data on the bleeding risk associated with uninterrupted anticoagulation and on the thromboembolic risk associated with anticoagulation interruption. This has led to several published clinical practice guidelines that endorse different management strategies. 4 5 6 7

Management of anticoagulation in patients undergoing colonoscopy and polypectomy will become more prevalent as the use of anticoagulation therapy increases with the aging population. 8 Direct oral anticoagulants (DOACs) have become the most prescribed class of oral anticoagulants because it is a more effective, safe, and convenient therapeutic alternative to vitamin K antagonists (VKA) in many clinical settings. 9 Although robust data on the bleeding risk of anticoagulated patients undergoing colonoscopy and polypectomy is not available, pooled data from cohort studies estimated that the risk of bleeding after elective endoscopic gastrointestinal procedures in patients on a DOAC is 3.1% when the DOAC was interrupted and 3.6% when the DOAC was continued without interruption. 4 Similarly, data from the Periprocedural Anticoagulation Use for Surgery Evaluation (PAUSE) trial, a prospective cohort study with a standardized protocol for DOAC interruption, reported a bleeding rate of 2.5% among the subgroup of patients undergoing endoscopic gastrointestinal procedures. 10 11

The recently published American College of Gastroenterology–Canadian Association of Gastroenterology Clinical Practice Guideline recommended continuation of VKA and temporary interruption of DOACs for patients undergoing colonoscopy and cold snare polypectomy of small polyps. 4 Due to insufficient evidence, the committee was unable to reach a recommendation on the timing of DOAC resumption; however, the accompanying dissemination tool indicated that the DOAC could be restarted the day after the colonoscopy in most patients. 12 This was a change from previous guidelines that recommended temporary interruption of VKAs for any polypectomy, with bridging anticoagulation for patients at high risk of thromboembolism; however, these published recommendations are based on low quality of evidence. 4 6 7 13 Organized CRC screening programs provide an opportunity to standardize and evaluate anticoagulation management in patients undergoing colonoscopy.

Given the high prevalence of patients on anticoagulation therapy and the low quality of evidence informing current guidelines, the British Columbia Colon Screening Program (BCCSP) in collaboration with the St. Paul's Hospital (SPH) Thrombosis Clinic created a multidisciplinary patient care pathway to standardize periprocedural anticoagulation management. A pilot project with a small group of selected patients was well-received by health care providers and patients. This was subsequently adopted as the standard of care in March 2022 for all anticoagulated patients referred for colonoscopy in the Vancouver region of the BCCSP. This novel patient care pathway provided an opportunity to conduct a real-world study to determine the incidence of adverse events after colonoscopy with polypectomy in the setting of highly standardized periprocedural anticoagulation management.

## Objective

The primary objective of this study is to determine the incidence of bleeding and thromboembolic complications among colon screening participants undergoing colonoscopy following a novel patient care pathway for standardized periprocedural anticoagulation management.

## Methods

A retrospective cohort study was conducted using available medical records and relevant imaging studies from the SPH Thrombosis Clinic and the BCCSP. Data were collected anonymously and recorded in the REDCap database by study team members. The first draft of the manuscript was written by the first, second, and last authors. All authors approved the final version of the manuscript.

### Participants

All BCCSP participants living in the Vancouver region referred for colonoscopy from March 1, 2022 to August 31, 2022 who were prescribed an oral anticoagulant were included. In the BCCSP, individuals 50 to 74 years of age undergo biennial FIT (OC-SENSOR, Eiken Chemical Limited, Tokyo, Japan; cutoff 10 µg globin/gram feces) are referred for colonoscopy to follow-up abnormal results. Precolonoscopy assessment is performed by a trained nurse (patient coordinator). The use of oral anticoagulants, including VKAs (warfarin) and DOACs (dabigatran, rivaroxaban, apixaban, and edoxaban), prompts a referral to the SPH Thrombosis Clinic. Prescription data were confirmed on PharmaNet, a centralized platform recording all prescriptions dispensed in pharmacies in British Columbia.

Colonoscopies were performed by local gastroenterologists and general surgeons. At the time of colonoscopy, the physician completes a standardized colonoscopy report form, which documents cecal intubation, the quality of the bowel preparation, any adverse events occurring during the colonoscopy, the polyp site and size, and method and completeness of polypectomy for each polyp detected. Fourteen days following colonoscopy, the patients are contacted by the patient coordinator and asked whether an unplanned medical event had occurred. If an unplanned event occurred, details regarding the event and any medical interventions are collected by the patient coordinator and submitted to the BCCSP. This process has been described previously. 3

### Standardized Periprocedural Anticoagulation Patient Care Pathway

**Fig. 1:**
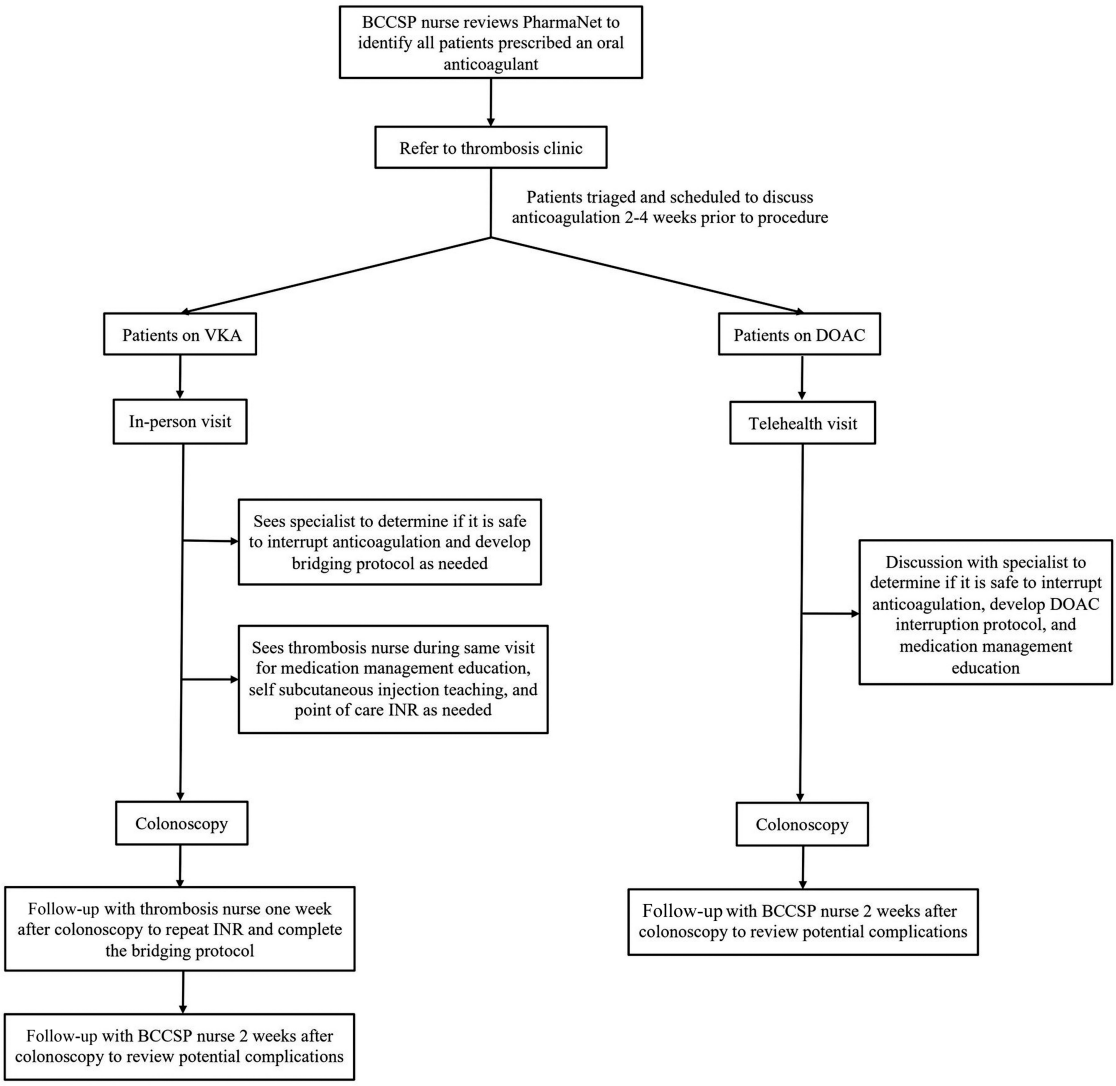
Interdisciplinary management pathway. PharmaNet is a province-wide database of all prescription medication dispensed by a licensed pharmacy in British Columbia. BCCSP, British Columbia Colon Screening Program; DOAC, direct oral anticoagulant; INR, international normalized ratio; VKA, vitamin K antagonist.

The periprocedural anticoagulation patient care pathway was implemented on March 1, 2022 and all patients on an oral anticoagulant were consecutively referred to the SPH Thrombosis Clinic for evaluation and management before the colonoscopy ( Fig. 1 ). Patients received comprehensive periprocedural anticoagulation assessment by a thrombosis medicine physician. Instructions on when to stop and when to resume the oral anticoagulant was provided to the patients before the colonoscopy. The timing of anticoagulation interruption and the need for bridging with LMWH were guided by clinical guideline recommendations, 14 15 but the final decisions were dependent on the thrombosis medicine physician's clinical judgement. DOAC perioperative assessments were performed virtually to minimize the time burden on the patients, but VKA perioperative assessments were conducted with in-person appointment due to the complexity in communicating multistep instructions and the need for international normalized ratio (INR) testing. Point of care INR testing was performed during the in-person appointment to avoid additional patient visits to the laboratory. During the same in-person appointment, the patients received an education session on VKA management by the thrombosis nurse. The thrombosis nurse managed the VKA dosing in the periprocedural period and then transferred the care back to the primary care provider once the colonoscopy was completed and the INR was stabilized. If the thrombosis medicine physician recommended bridging with LMWH, the thrombosis nurse provided patient education on subcutaneous self-injection. The prescription for LMWH was provided by the thrombosis medicine physician. During the 14-day telephone follow-up, the BCCSP patient coordinator confirmed the resumption of oral anticoagulant.

### Outcomes

The primary outcomes of interest were bleeding events and thromboembolic events from the time of oral anticoagulant interruption until 14 days after the colonoscopy. The outcome was ascertained by the BCCSP patient coordinator and documented using standardized forms. All unplanned medical events were reviewed and adjudicated by two thrombosis medicine physicians on the study team. Bleeding events were defined by the International Society on Thrombosis and Hemostasis (ISTH) definition of major bleeding (fatal bleeding, symptomatic bleeding in a critical area or organ, bleeding resulting in a decrease in hemoglobin of ≥ 20 g/L, or leading to transfusion of ≥ 2 units of blood). 16

Thromboembolic events included arterial thromboembolic events (ischemic stroke, transient ischemic attack, and systemic arterial embolism) and venous thromboembolism. The secondary clinical outcomes were clinically relevant nonmajor bleeding (ISTH definition), minor bleeding (ISTH definition), acute coronary syndrome, emergency room visit, hospital admission, and death due to any cause.

### Statistical Analysis

Demographic characteristics of patients and colonoscopy findings were summarized with descriptive statistics. The incidence rates for all major bleeding and thromboembolism were reported in subgroups of patients on different oral anticoagulants.

## Results

**Fig. 2:**
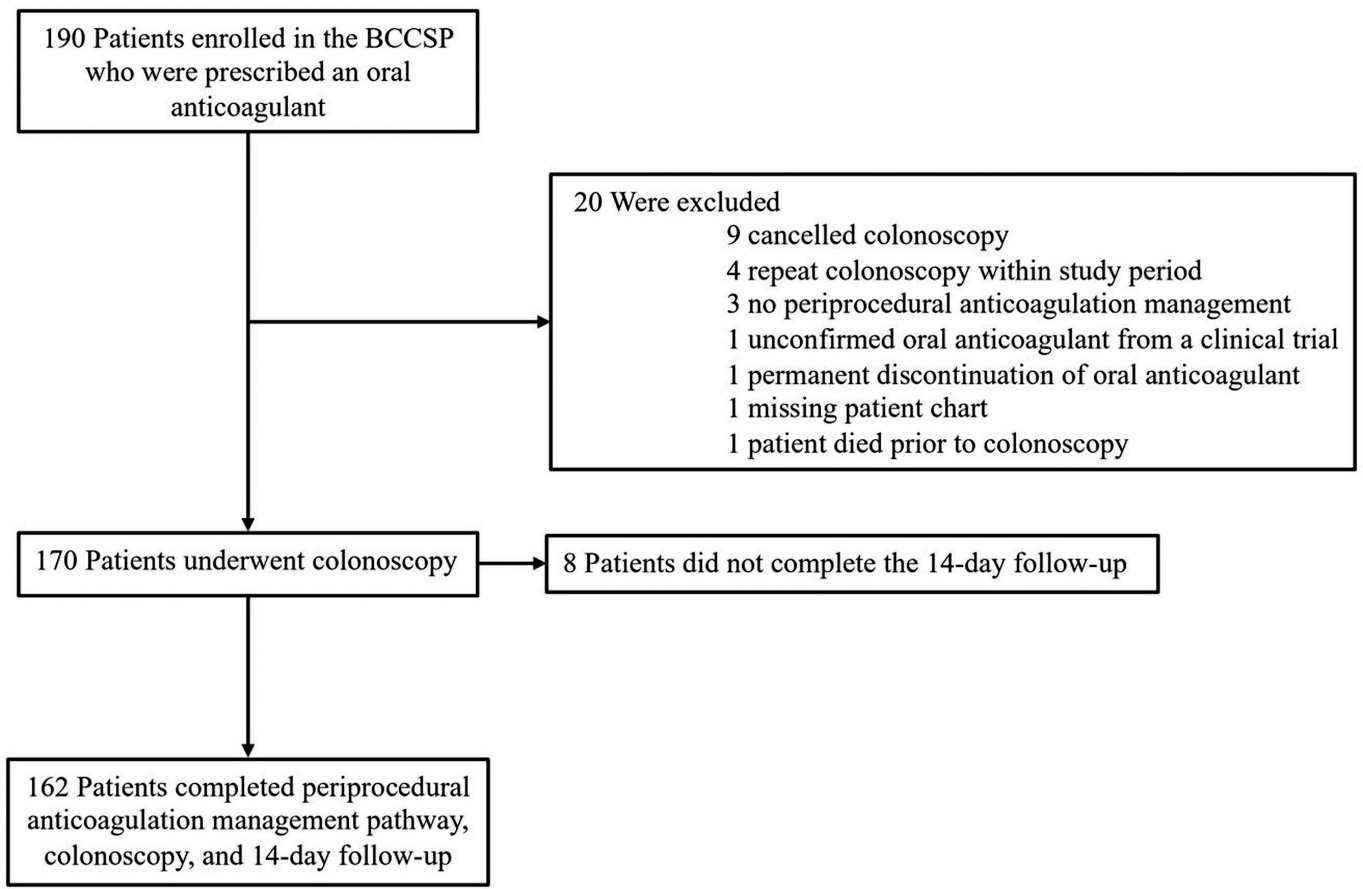
Participant flow diagram. BCCSP, British Columbia Colon Screening Program.

Over the 6-month study period (March 1, 2022 to August 31, 2022), 190 BCCSP patients referred for colonoscopy were prescribed an oral anticoagulant. Twenty patients were excluded, and eight patients were lost to follow-up ( Fig. 2 ). The patients had a mean (standard deviation [SD]) age of 67 (6.3) years and 73.3% were male. Of the included patients, 79 (46.5%) were on rivaroxaban, 61 (35.9%) were on apixaban, 10 (5.9%) were on dabigatran, 1 (0.6%) were on edoxaban, and 19 (11.2%) were on warfarin. A total of 7.6% of the patients had concomitant use of antiplatelet therapy. The indications for anticoagulation include atrial fibrillation in 135 patients, venous thromboembolism in 31 patients, and mechanical heart valve in 5 patients ( Table 1 ). Some patients had more than one indication for anticoagulation. The atrial fibrillation patients had a mean (SD) CHADS65 score of 2.2 (1.1).

**Table 1:** Baseline patient characteristics

Variable	*n* = 170	
Age, mean (SD) in years	66.9	(6.3)
Male (%)	124	(72.9)
Weight, mean (SD), kg	88.9	(22.6)
Comorbidities, *n* (%)		
Heart failure	18	(10.6)
Hypertension	98	(57.6)
Diabetes	41	(24.1)
Cerebral arterial disease	29	(17.1)
Bioprosthetic heart valve	5	(2.9)
Severe mitral stenosis	4	(2.4)
Coronary artery disease	32	(18.8)
Chronic kidney disease	33	(19.4)
Mild (CrCl 50–70 mL/min)	23	
Moderate (CrCl 30–49 mL/min)	10	
Severe (CrCl < 30 mL/min)	0	
Inherited thrombophilia	5	(2.9)
Antiphospholipid syndrome	1	(0.6)
Active cancer	3	(1.8)
Gastrointestinal bleed history	11	(6.5)
Lab value, mean (SD)		
Hemoglobin	140.8	(18.4)
Platelet	233.7	(69.2)
Creatinine	88.7	(21.7)
Indication for anticoagulation, *n*		
Atrial fibrillation	135	
Mean CHADS65 score	2.2	
Venous thromboembolism	31	
Mechanical mitral valve	2	
Mechanical aortic valve	3	
Anticoagulant use, *n* (%)		
Rivaroxaban	79	(46.5)
Apixaban	61	(35.9)
Dabigatran	10	(5.9)
Edoxaban	1	(0.6)
Warfarin	19	(11.2)
Concurrent antiplatelet use, n (%)		
Aspirin	11	(6.5)
P2Y12 inhibitor	2	(1.2)
Other medication use, *n* (%)		
Selective serotonin reuptake inhibitor	9	(5.3)
CYP 3A4 inhibitor	13	(7.6)
CYP 3A4 inducer	1	(0.6)

Abbreviations: CrCl, creatinine clearance; SD, standard deviation.

### Periprocedural Anticoagulation Management

DOAC interruption intervals are reported in Table 2 . A total of 63.2% of patients on VKA required bridging with LMWH. In the preprocedural period, 91.7% of bridging used therapeutic dose LMWH. In the postprocedural period, 75.0% of bridging used therapeutic dose LMWH ( Table 3 ).

**Table 2:** Perioperative direct oral anticoagulant management

Preprocedural DOAC management, *n* (%)		
Last dose day −3	146	(96.7)
Last dose day −2	5	(3.3)
Postprocedural DOAC management, *n* (%)		
Resumed day +1	70	(46.4)
Resumed day +2	80	(53.0)
Resumed day > 2	1	(0.7)

Abbreviation: DOAC, direct oral anticoagulant.

**Table 3:** Perioperative vitamin K antagonist management

Bridging vs. no bridging	*n* = 19
Uninterrupted VKA	1
Interrupted VKA with no bridging	6
Interrupted VKA with bridging	12
Preprocedural bridging	
Preop bridge with therapeutic low molecular weight heparin	11
Preop bridge with nontherapeutic low molecular weight heparin	0
No preop bridging	1
Postprocedural bridging	
Postop bridge with therapeutic low molecular weight heparin	9
Postop bridge with nontherapeutic low molecular weight heparin	3

Abbreviations: Postop, postoperative; preop, preoperative; VKA, vitamin K antagonist.

### Colonoscopy and Polypectomy Characteristics

A total of 170 colonoscopies were performed. Two colonoscopy reports were missing. In the remaining 168 colonoscopy reports, a few reports had missing information on the location, size, mode of removal, and morphology of the polyps. The bowel preparation was adequate in 158 (94%). A total of 133 (79%) patients underwent at least one polypectomy with a mean number of polypectomies per colonoscopy of 2.6. A total of 338 polyps were removed and the polyp characteristics are shown in Table 4 .

**Table 4:** Polyp characteristics

Polyp characteristics	Number (%)	
Location		
Ileum	1	(0.3)
Cecum	30	(8.9)
Ascending colon	80	(23.7)
Transverse colon	76	(22.5)
Descending colon	42	(12.4)
Sigmoid colon	73	(21.6)
Rectum	36	(10.7)
Size		
≤5 mm	196	(58.0)
6–9 mm	110	(32.5)
10–19 mm	28	(8.3)
≥20 mm	2	(0.6)
Unknown	2	(0.6)
Morphology		
Sessile	275	(81.4)
Pedunculated	31	(9.2)
Flat	15	(4.4)
Other	9	(2.7)
Unknown	8	(2.4)
Mode of removal		
Cold snare	274	(81.1)
Hot snare	34	(10.1)
Biopsy forceps	28	(8.3)
Unknown	2	(0.6)

### Study Outcomes

With regard to the primary outcomes, one (0.6%) patient had a major bleeding event occurring 6 days after colonoscopy and one (0.6%) patient had an arterial thromboembolic event postcolonoscopy.

In terms of secondary outcomes, there were no reported clinically relevant nonmajor bleeding or minor bleeding. One patient had acute coronary syndrome 8 days after colonoscopy. Five patients had an unplanned event requiring emergency department treatment. Among these emergency department visits, two resulted in hospital admissions. There were no reported deaths.

## Discussion

The rates of major bleeding, arterial thromboembolism, and venous thromboembolism after colonoscopy with polypectomy were low (0.6, 0.6, and 0%, respectively) in our real-world cohort of patients who were on an oral anticoagulant and received periprocedural anticoagulation management in a standardized patient care pathway by a multidisciplinary team. The pathway offered patients with high-value, comprehensive care by multiple expert providers in fewer in-person medical visits.

The major bleeding rate in the present study was similar to the 0.9% major bleeding rate reported from the PAUSE trial data. 11 Other cohorts where postprocedure bleeding ranged from 2.5 to 3.6%. 4 Over 90% of the patients in our cohort had small polyps (less than 10 mm in diameter), and over 80% underwent cold snare polypectomy. Two previously published randomized trials support the use of cold snare polypectomy in anticoagulated patients. Takeuchi et al compared uninterrupted oral anticoagulants and cold snare polypectomy to interrupted oral anticoagulants and hot snare polypectomy in patients undergoing colonoscopy and removal of polyps up to 10 mm in diameter. 17 The cold snare polypectomy strategy was noninferior with 4.7% (95% confidence interval [CI]: 0.2–9.2%) of patients sustaining postpolypectomy bleeding, whereas 12% (95% CI: 5.0–19.1%) of patients who underwent hot snare polypectomy experienced bleeding. Second, Horiuchi et al randomized patients on continuous VKA to cold or hot snare polypectomy of small polyps. The cold snare polypectomy arm had a significantly lower rate of immediate and delayed bleeding. 18

In the current study, of the 338 polyps resected, only one patient had a major bleeding event, following removal of a large lesion. This patient had held rivaroxaban 2 days prior to colonoscopy and resumed 2 days following the procedure. A 3-cm lateral spreading tumor of the ascending colon was removed in multiple pieces by endoscopic mucosal resection using a combination of hot and cold snare polypectomy. Intraprocedural bleeding was treated with cauterization with satisfactory hemostasis. No clipping was used. The patient was managed conservatively with blood transfusion in the emergency department and interrupted rivaroxaban therapy for an additional 7 days. The patient did not require hospital admission or repeat endoscopic procedure. This case illustrated that endoscopic mucosal resection of large polyps may be associated with postpolypectomy bleeding and delayed resumption of DOACs should be considered. In the PAUSE trial protocol, patients undergoing high risk bleeding procedures resumed the DOAC 48 to 72 hours after the procedure. This is consistent with findings from other studies. 11 19 The patients with a low thromboembolic risk, including patients with atrial fibrillation with a CHADS65 score < 4 and patients with venous thromboembolism treated for more than 3-6 months, will particularly benefit from delayed resumption of oral anticoagulant after resection of large lesions.

The thromboembolic event rate of 0.6% observed in the current study was similar to the arterial thromboembolic event rate of 0.7% among patients in the PAUSE trial undergoing endoscopic gastrointestinal procedures. 11 For this patient in our study, the indication for anticoagulation was atrial fibrillation with a CHADS65 score of 1. In addition, the patient had a history of type 1 aortic dissection 8 months prior to the colonoscopy, which was managed with ascending aorta and aortic arch replacement and bioprosthetic aortic valve replacement. Rivaroxaban was held 2 days prior to colonoscopy and resumed 2 days following colonoscopy. Two polyps were removed: a 6-mm pedunculated polyp in the descending colon was resected with hot snare polypectomy and a prophylactic hemoclip was placed on the base and in the transverse colon, a 3-mm polyp was resected with cold snare polypectomy. Three days following colonoscopy, the patient was diagnosed with a transient ischemic attack in the emergency department and ultimately did not require hospital admission. This event may have been potentially preventable if the DOAC was not interrupted. However, it is clinically challenging to identify patients who will most likely benefit from polypectomy with uninterrupted anticoagulation until we have more data and experience with this strategy, particularly with patients on a DOAC. Importantly, the patient's preference is another important factor in deciding on the strategy.

Nevertheless, we demonstrated that a standardized patient care pathway with collaboration between different specialists involved in the patient's CRC screening journey can minimize adverse events related to anticoagulant use in the real world. As there is variability in guideline recommendations and clinical adherence to institutional guidelines regarding periprocedural anticoagulation management during colonoscopy, 20 our standardized, multidisciplinary pathway with appropriate follow-up allows consistency in evaluating safety outcomes and determining the most optimal anticoagulation management for each individual patient. A standardized pathway is especially important given that the BCCSP covers many different sites across a large geographic area, and therefore, inconsistency in local adherence and policies can be a large issue. Clear communication between the different health care providers and the patients is essential as the timing of anticoagulation resumption depends on the findings on the colonoscopy. Another strength to this standardized pathway is the early identification of postprocedure thrombotic and bleeding complications, which allows appropriate specialists (gastroenterologists, thrombosis specialists, etc.) to see the patient in a timely manner as the team is already involved in the patient's care prior to the procedure. Our findings suggested that most patients with atrial fibrillation and venous thromboembolism can safely interrupt oral anticoagulant for polypectomy because the rate of arterial and venous thromboembolism is low. However, a small group of patients with a very high thromboembolic risk, such as those with mechanical heart valves or acute venous thromboembolism within the prior 3 months, may benefit from polypectomy with uninterrupted oral anticoagulant. It is reassuring that a recent randomized control trial demonstrated that the rate of major bleeding after cold snare polypectomy with uninterrupted VKA is low. 21 To our knowledge, there are limited data on the bleeding risk after polypectomy with uninterrupted DOAC.

Further studies will evaluate this standardized, multidisciplinary pathway over a longer time period, with a larger cohort through expanding this pathway to more health authorities and institutions. These future analyses will allow for cost–benefit analyses; we hypothesize that this standardized pathway among multiple disciplines (e.g., colon screening program nurses, thrombosis physicians, gastroenterologists) will reduce the number of delayed and canceled procedures and address postprocedure complications adequately, overall reducing system costs due to loss to follow-up, and lack of standardized anticoagulation management.

This study has important limitations. First, this was a retrospective study, so some relevant clinical information may be missing. Second, the sample size of 162 was modest, with patients on VKAs underrepresented. We decided on the 6-month study period to identify potential areas for improvement in the standardized patient care pathway early and implement adjustments as needed sooner than later. Third, the event rates were low preventing further investigation of putative variables associated with bleeding or thromboembolism. Fourth, the follow-up period of 14-day postcolonoscopy may miss a minority of delayed postpolypectomy bleeding or thromboembolic events. 22 The PAUSE trial followed participants for 30 days and the thromboembolic events occurred at a mean of 24 days. 10 Since this is a retrospective study, the data depend on the existing documentation and the 14-day follow-up has been the local practice of the BCCSP for years.

The strength of this study is the real-world environment enabling the results to be generalized to other screening programs. Our standardized periprocedural patient care pathway ensured consistency in anticoagulation management, and it minimized the number of patients who were lost to follow-up.

## Conclusion

This study showed that a standardized patient care pathway for periprocedural anticoagulation management with multidisciplinary collaboration was associated with low rates of major bleeding and thromboembolism after colonoscopy with polypectomy. Future studies assessing a strategy of uninterrupted VKA or DOAC with cold snare polypectomy are needed.
